# Rapid bioassay-guided screening of toxic substances in vegetable oils that shorten the life of SHRSP rats

**DOI:** 10.1186/1476-511X-9-13

**Published:** 2010-02-02

**Authors:** Sunil Ratnayake, Paul Lewandowski

**Affiliations:** 1 Molecular Nutrition Unit, School of Medicine, Deakin University, Pigdons Road, Waurn Ponds VIC 3217, Australia

## Abstract

It has been consistently reported that vegetable oils including canola oil have a life shortening effect in Stroke-Prone Spontaneously Hypertensive Rats (SHRSP) and this toxic effect is not due to the fatty acid composition of the oil. Although it is possible that the phytosterol content or type of phytosterol present in vegetable oils may play some role in the life shortening effect observed in SHRSP rats this is still not completely resolved. Furthermore supercritical CO_2 _fractionation of canola oil with subsequent testing in SHRSP rats identified safe and toxic fractions however, the compounds responsible for life shortening effect were not characterised. The conventional approach to screen toxic substances in oils using rats takes more than six months and involves large number of animals. In this article we describe how rapid bioassay-guided screening could be used to identify toxic substances derived from vegetable oils and/or processed foods fortified with vegetable oils. The technique incorporates sequential fractionation of oils/processed foods and subsequent treatment of human cell lines that can be used in place of animal studies to determine cytotoxicity of the fractions with structural elucidation of compounds of interest determined *via *HPLC-MS and GC-MS. The rapid bioassay-guided screening proposed would require two weeks to test multiple fractions from oils, compared with six months if animal experiments were used to screen toxic effects. Fractionation of oil before bio-assay enhances the effectiveness of the detection of active compounds as fractionation increases the relative concentration of minor components.

## Background

Fat is an important part of the diet that provides the greatest amount of energy per gram of any food. However excessive consumption of saturated fat may raise blood cholesterol levels and lead to an increased risk for coronary heart disease. Fatty acid composition of fats and oils also has a major impact on human health. Factors such as low saturated fatty acids, high unsaturated fatty acid and relatively low ω6/ω3 polyunsaturated fatty acid ratio have been associated with favourable health benefits. Among the oils available for human consumption in today's market, canola oil has the properties that would be considered as beneficial having very low saturated fats (7%), relatively high in monounsaturated fat (61%) with moderate levels (22%) of omega-6 (ω-6); linoleic acid and a relatively high amount (11%) of omega-3 (ω3); alpha-linolenic acid. With an ever increasing use of canola oil in processed foods, fast foods and home cooked meals in countries such as Australia, Canada and Japan the amount of canola oil consumed by some individuals is much higher than in previous decades.

Since 1996 a number of studies have been conducted which have compared the effects of various vegetable oils on the lifespan of Stroke-Prone Spontaneously Hypertensive Rats (SHRSP) [1-3]. It has been consistently reported that canola, corn and olive oil have life shortening effects in SHRSP rats when the oil is added to the rats' standard diet, compared with other vegetable oils such as soybean, safflower and perilla. Typical results with canola oil indicate that the rats die within 110 days compared with 160 days for rats fed the other oils such as soybean oil [1-3]. In these previous experiments the diets consumed by the rats were identical except for the oil being tested that provided 29% of energy. Initially, it was felt that the fatty acids in the oils were responsible for the differences in longevity. Soybean oil was relatively safe in this model but partially-hydrogenated soybean oil shortened survival time [4]. Free fatty acid (FFA) fractions obtained from the triacylglycerols (TAG) after hydrolysis of canola oil and hydrogenated soybean oil exhibited no or decreased life shortening activity [3]. Confirming these observations, Ratnayake *et al*. [5,6] added high oleic-safflower oil and high-oleic sunflower oil to the list of oils those with no life shortening activity in SHRSP rats. Based on the lack of life shortening effect from this range of oils it was concluded that the life shortening effect was not accounted for by the difference in their fatty acid compositions, hence the presence in these oils of minor components to stimulate the onset of stroke and shorten the survival was presumed.

The next oil component to be investigated for a life shortening effect was plant sterols (phytosterols). SHRSP rats fed with canola oil and corn oil which contain higher amounts of natural plant sterols than other vegetable oils showed lower survival time compared to soy oil [6]. The mean survival time of SHRSP rats fed soybean oil fortified with plant sterols isolated from canola oil was reduced compared to unfortified soybean oil fed to SHRSP rats. Furthermore margarine fortified with plant sterols or stanols reduced the life span of SHRSP rats [7]. However, in contrast olive oil, that has the lowest phytosterol content of oils studied to date, showed the greatest survival shortening activity [6] suggesting that phytosterols are not the only factor responsible for the life shortening effect observed in SHRSP rats. The SHRSP rat is commonly used as a model of hypertension and cardiovascular disease in humans; developing cerebral hemorrhagic stroke. SHRSP rats fed high plant sterol diets had elevated levels of phytosterols in plasma, red blood cells, liver and kidney compared with rats fed a low phytosterol diet suggesting that the accumulation of plant sterols may contribute to the life shortening effect observed in SHRSP rats [6]. Hyperabsorption and accumulation of dietary plant sterols in SHRSP rats is attributed to a mutation in the ABCG5 or ABCG8 genes [8]. In contrast, humans retain low levels of plant sterols (1-5% of total sterols) accept for phytosterolemic patients that retain 15-20% of dietary plant sterols [8]. In another study, the presence of factors other than phytosterols had to be considered because the phytosterol composition was very similar for high oleic type and high-linoleic type of safflower oil but survival rates were very different between these two oils, and between soybean oil and hydrogenated soybean oil [9]. Furthermore, the addition of 5 times more phytosterol to soy oil compared to that of canola oil was reported to be necessary to reproduce the life shortening activity equivalent to canola oil [10]. Such studies were not able to conclude that the phytosterols were the only component responsible for the life shortening effects observed with vegetable oils including canola oil in SHRSP rats; it is possible that the phytosterol content and type of phytosterol might play some role in the life shortening effect although this is still not completely resolved.

Another approach to explore toxic substance in canola oil was reported by Ohara *et al*. [11] that involved fractionated of canola oil using super critical CO_2 _separation of the oil with subsequent treatment testing in SHRSP rats. This investigation only produced three crude fractions from canola oil rich in campesterol and β-sitosterol with the lesser amounts of brassicasterol and stigmasterol and failed to clearly separate the causative substances into a specific fraction and was unable to characterise the compounds responsible for life shortening of the rats.

While it is clear that canola oil, corn oil and olive oil have a life shortening effect in the SHRSP rat the question must be asked, is it a strain specific effect or is the consumption of these vegetable oils as the primary dietary fat source also pathogenic in other rat strains or human populations that consume large amount of one of these oils oil? When canola oil was the only dietary fat fed to normotensive Wistar Kyoto rats, the strain the SHRSP was original derived from, a significant increase in systolic blood pressure after five weeks of canola oil feeding was observed and persisted until the conclusion of the trial at week 13, compared to soybean oil fed rats [12]. In addition to this increase in blood pressure the canola oil fed group showed a higher density of neutrophils and a decrease in the activity of the antioxidant enzymes catalase and superoxide dismutase together with an enhanced production of NADPH [12]. More recently, spontaneously hypertensive rats (SHR) rats were used to study the life shortening effect of canola oil to avoid possible bias due to the irregular deaths by stroke seen in SHRSP rats [13]. These investigations in SHR showed that elevated plasma lipids and glucose-6-phosphate dehydrogenase (G6PD) activation in the liver and erythrocyte in SHR fed canola oil compared to that of fed soy oil with the significant vascular lesions in the kidney of canola oil fed group compared to soy oil group. Furthermore Okuyama *et al*. [14] reported that the low ratio of ω6/ω3 in perilla oil (1:4) and fish oil extended the lifespan of SHRSP rats compared that of with safflower oil (1:20) and soybean oil (1:10) but canola oil with relatively low ω6/ω3 ratio (1:2.5) shortened the survival of this strain as compared with soybean oil [1,2].

In human populations there is only one published incidence of canola oil being associated with the development of disease [15] and this due was to manufacturing error when producing canola oil. However the consumption of canola oil by many individuals is much higher than in previous decades due to increased use of canola in processed foods, fast foods and home cooked meals than other oils available in today's market. Irrespective of whether vegetable oils such as canola oil have a life shortening effect in humans similar to SHRSP rats, an interesting scientific paradigm is still present warranting further investigation to identify the components of the oils responsible for shortening life. Once identified the relevance of these compounds toxic to SHRSP rats to human health can more clearly be studied.

In summary it has been consistently reported that some oils including canola oil have life shortening effects in SHRSP rats and this effect is not due to the fatty acid composition of the oil; it is possible that the phytosterol content and type of phytosterol might play some role in the life shortening effect although this is still not completely resolved. The conventional approach to screen toxic substances in vegetable oils using animal models takes more than six months and involves a large number of animals depending on the number of oil fractions to be tested. In this report we explain an alternative rapid bioassay-guided screening approach to elucidate biologically active substances in oils using human cell lines in place of animal models.

## Testing hypothesis

Naturally produced oils are a complex mixture of non polar TAG, a large number of phyto compounds such as phenolic compounds including phenolic acids, flavonoids, tannins, sterols and oxidised TAG [6,16-18]. The use of rapid bioassay-guided screening technique to screen the crude extracts or semi-pure fractions of natural products allows the detection of biologically active compounds. Fractionation of crude extracts of natural product mixtures before bio-assay may enhance the effectiveness of the detection of active compounds as fractionation reduces the inference of the compounds with one another. Reduced interference by the simplification of the mixtures increases the relative concentrations of minor components and increases the opportunity to uncover novel biologically active metabolites [19].

The conventional approach to screen toxic substances in vegetable oils using rats takes extended period of time, is a costly exercise and requires large number of animals. Fractionation produces numerous fractions according to polarity and molecular weight, and it would not be feasible to use animals to test the biologically active compounds in each fraction on such a large scale and also the production of suitable quantities of each fraction to be incorporated to the animal diet is limiting. This incites the necessity of develop an alternative approach to screen toxic substances in vegetable oils. In addition the proposed technique significantly reduces the cost and the time required to screen for toxic substances. Employing a cytotoxicity bio-assay to test five fractions would only take two weeks compared with six months if animal experiments were used to screen toxic effects. In this bio-assay, 96-well microtiter culture plates would be seeded at 1 × 10^4 ^human cells/well in media and incubated overnight before treatment. Each fraction would be tested at five different concentrations ranging 50-1800 μg/ml in triplicates.

The non polar components mostly TAG of the oils can be separated from polar compounds in vegetable oils by chromatography using silica gel column [16,17,20]. Polar compounds include sterols, triterpenic acids, FFA and modified TAG such as thermally modified polymers, oxidized monomers and diacylglycerols (DAG) [21]. The polar fraction of the oil can be further fractionated according to the molecular weight of compounds by chromatography using a stationary column packed with Sephadex LH-20 and ethanol as an elution solvent [22].

The proposed system would carry out sequential fractionation of vegetable oils according to the polarity and molecular weight. Subsequent treatment of human cell lines would determine cytotoxicity effects of the fractions with structural elucidation of compounds of interest determined *via *High Performance Liquid Chromatography-Mass Spectroscopy (HPLC-MS) and/or Gas Chromatography-Mass Spectroscopy (GC-MS). The proposed approach would be able to conquer most of the constraints of the conventional rat based approach.

## Implication of the hypothesis

The use of canola oil by food manufactures, commercial users and consumers has increased over recent years, primarily due to the marketing image that canola oil is a healthy alternative. Since the role of phytosterols on survival time in SHRSP rats is still not completely resolved other minor compounds in dietary oils may have toxic effects on rats and humans. Even though humans absorb less plant sterols than SHRSP rats, individuals that consume increased amounts of canola oil for lengthy period may be at risk of adverse health outcomes, due to phytosterols or other oil components. Rapid screening for toxic substances responsible for the life shortening effects of canola oil in the SHRSP rat is of particular importance because it is a potentially challenging issue for canola farmers and consumers of canola oil i.e. what can be done to remove the toxic compound(s) from canola oil. Once toxic compounds have been identified further studies would be carried out to remove toxic compounds via modification to oil processing methods and would resolve existing doubts as to the safety of consuming canola oil by humans. Authors would like to emphasize that the proposed bio assay-guided screening technique is cost effective and quick method to identify supplements or extracts that are bioactive and provide an insight as the composition of the bioactive compounds(s) prior to undertaking further time consuming and expensive animal or clinical studies.

## List of abbreviation

(SHRSP): Stroke-Prone Spontaneously Hypertensive Rats; (SHR): spontaneously hypertensive rats; (ω6): omega-6; (ω3): omega-3; (G6PD): glucose-6-phosphate dehydrogenase; (TAG): triacylglycerols; (FFA): Free fatty acids; (DAG): diacylglycerols; (HPLC-MS): High Performance Liquid Chromatography-Mass Spectroscopy; (GC-MS): Gas Chromatography-Mass Spectroscopy.

## Competing interests

The authors declare that they have no competing interests.

## Authors' contributions

SR and PAL contributed equally to the manuscript and have both read and approved the final manuscript.
